# Real-time fMRI neurofeedback boosts heartbeat perception by modulating insula activation pattern during interoceptive attention

**DOI:** 10.1162/IMAG.a.142

**Published:** 2025-09-09

**Authors:** Yusuke Haruki, Yuxiang Yang, Keisuke Suzuki, Hiroshi Imamizu, Kenji Ogawa

**Affiliations:** Department of Psychology, Graduate School of Humanities and Human Sciences, Hokkaido University, Sapporo, Japan; Japan Society for the Promotion of Science, Tokyo, Japan; Center for Human Nature, Artificial Intelligence, and Neuroscience (CHAIN), Hokkaido University, Sapporo, Japan; Department of Life Sciences, Graduate School of Arts and Sciences, The University of Tokyo, Tokyo, Japan; Key Laboratory of Adolescent Cyberpsychology and Behavior (CCNU), Ministry of Education, Wuhan, China; Key Laboratory of Human Development and Mental Health of Hubei Province, School of Psychology, Central China Normal University, Wuhan, China; Department of Psychology, Graduate School of Humanities and Sociology, The University of Tokyo, Tokyo, Japan; Cognitive Mechanisms Laboratories, Advanced Telecommunications Research Institute International, Kyoto, Japan

**Keywords:** neurofeedback, fMRI, heartbeat perception, attention, interoceptive awareness, insula

## Abstract

Real-time fMRI neurofeedback (NF) has emerged as a promising method for enabling individuals to modulate specific brain regions and, consequently, their behavioural outcomes. This study examined whether the NF targeting the right insula could improve heartbeat perception ability and influence emotional response to negatively valenced stimuli, by training participants to modulate the brain activation associated with interoceptive (heartbeat-focused) and exteroceptive (visual-focused) attention. Fifty-four participants underwent a single ~40-minute NF session with contingent (NF group, n = 28) or non-contingent (Sham group, n = 26) feedback, with heartbeat perception and emotional appraisal assessed pre- and post-training. The NF group demonstrated significant improvements in heartbeat perception, with individual learning effects in neuromodulation predicting the behavioural gains. However, group-level NF scores did not differ significantly, likely reflecting variability in learnability. Despite improvements in heartbeat perception, NF training did not modulate emotional responses at either the behavioural or neural level, suggesting that targeting the insula alone is insufficient to alter affective processing within a single session. These findings provide evidence that NF can enhance heartbeat perception through targeted neuromodulation in the insular cortex.

## 1 Introduction

Interoceptive processing involves the detection, integration, and regulation of internal bodily signals that convey visceral and metabolic states (Berntson & Khalsa, 2021; Petzschner et al., 2021). These continuous signals are essential for maintaining homeostasis and guiding adaptive behaviour while also playing a central role in emotional experiences, decision making, and sense of self (Blanke et al., 2015; Greenwood & Garfinkel, 2024; Seth & Tsakiris, 2018). However, interoceptive signals are often less salient than exteroceptive ones (e.g., vision and hearing), leading to substantial inter-individual variability in interoceptive awareness—the conscious perception of internal bodily states (Haruki et al., 2025; Khalsa et al., 2018; Murphy et al., 2018). Therefore, enhancing interoceptive awareness has been a focus of various interventions, particularly mindfulness and contemplative practices, which have been shown to improve bodily awareness, stress resilience, and emotional regulation (Bornemann & Singer, 2017; Fischer et al., 2017; Khoury et al., 2018; Riva et al., 2021). These approaches, however, usually require extensive and long-term training, limiting their applicability in both research and clinical settings due to issues such as participant dropout.

Real-time fMRI neurofeedback (NF) has emerged as a powerful technique for training individuals to voluntarily regulate neural activity in specific brain regions by providing them with real-time feedback on their own brain signals. By making neural activity observable, NF enables participants to develop control strategies that influence cognitive, affective, and sensorimotor processes (Pindi et al., 2022; Sulzer et al., 2024). This approach has been successfully applied in multiple domains, including emotion regulation (Caria et al., 2007; Herwig et al., 2019), pain suppression (Emmert, Breimhorst, et al., 2017), and motor learning (Sampaio-Baptista et al., 2021; Yang et al., 2021). A particularly promising application of NF is the training of interoceptive processing, leveraging the intrinsic brain–body connection to promote integrated psychophysiological states similar to those cultivated through contemplative practices (Treves et al., 2024; Wiebking & Northoff, 2014). However, despite its promise, interoceptive-focused NF remains underexplored, and few studies have demonstrated clear behavioural improvements following NF-driven neuromodulation.

Targeting the insular cortex with fMRI NF presents a promising approach of refining interoceptive processing, as the insula serves as a crucial hub for integrating internal bodily signals (Candia-Rivera et al., 2024; Craig, 2009; Sammons et al., 2024; Wang & Chang, 2024). This integration extends beyond interoceptive processing, as the insula also plays a key role in exteroceptive processing, linking visual or auditory saliency to ongoing bodily states (Gogolla, 2017; Uddin, 2015). Given that interoceptive signals are often suppressed by exteroceptive information (Ainley et al., 2016; Haruki & Ogawa, 2024), individuals may struggle to prioritise allocating attention to internal bodily sensations. Thus, training individuals to enhance interoceptive attention through NF could strengthen their ability to shift focus towards internal bodily signals, potentially improving behavioural interoceptive awareness. However, prior research has reported somewhat challenging outcomes: Zhang and colleagues reported that participants receiving continuous feedback based on left insular BOLD activity were able to increase their activation compared with a S-controlled group during heartbeat attention. Yet, they did not exhibit significant across-session learning of neuromodulation or behavioural improvements in heartbeat perception (Zhang et al., 2023). These findings highlight the need for refined NF paradigms that not only modulate insular activation but also translate into tangible behavioural outcomes.

Our primary objective is to test whether a single ~40-minute session of real-time fMRI NF can induce measurable learning effects in neuromodulation and behavioural improvements by dynamically shifting between interoceptive and exteroceptive attention. Our training involved segregating attentional states between interoceptive (heartbeat-focused) and exteroceptive (visual-focused) in the right insula, allowing participants to learn to reproduce distinct activation patterns associated with each state. This region was specifically targeted due to its well-established role in cortical interoceptive processing (Craig, 2009; Critchley et al., 2004; Haruki & Ogawa, 2021) and in general exteroceptive attention (Eckert et al., 2009), whereas a left-dominant analogue has not been established. To assess the impact of NF training, we measured interoceptive accuracy (i.e., abilities to correctly perceive interoceptive signals) using a heartbeat counting task before and after NF training, alongside an emotional appraisal task to explore potential affective changes. We hypothesise that participants receiving real-time NF contingent on their own brain activity will demonstrate significantly greater within-session learning and post-training enhancements in interoceptive accuracy and emotional regulation compared with a sham-feedback control group. Through this approach, we aim to rapidly recalibrate brain–body interactions and elucidate the mechanisms underlying NF-induced behavioural changes, potentially mirroring the benefits associated with long-term mindfulness training.

## 2 Materials and Methods

### 2.1 Participants

We used a single-blind and sham controlled experimental design. A total of 54 participants (32 males, 22 females; mean age = 23.04 years, SD = 2.65) were recruited and allocated to one of two groups. The NF group (n = 28; 16 males, 12 females) received contingent feedback during the task, whereas the Sham group (n = 26; 16 males, 10 females) received feedback totally unrelated to their brain activations. The sample size was determined based on a systematic review suggesting that 23 participants per group would be required for an NF training to detect medium-to-large effect sizes with sufficient statistical power (Tursic et al., 2020). Participants were recruited sequentially, with the first 26 assigned to the NF group, the next 26 to the Sham group. Towards the end of the data collection period, due to the availability of additional resources, two final participants were recruited and included in the NF group to increase its sample size. Although participants were assigned in a fixed order, there was a significant difference in age between groups: the NF group was older (mean = 24.29, SD = 2.57) than the Sham group (mean = 21.69, SD = 2.04), *t*_52_ = 4.094, *p* < .001. Each participant completed a single 2-hour experimental session and received 4000 JPY as compensation. Participation was voluntary, and no further incentives (e.g., course credit) were provided. Written informed consent was obtained from all individuals before commencing the study, in accordance with the Declaration of Helsinki. The ethics committee of the Center for Experimental Research in Social Sciences at Hokkaido University reviewed and approved all experimental procedures in advance.

### 2.2 Experiment procedure

**Fig. 1.**
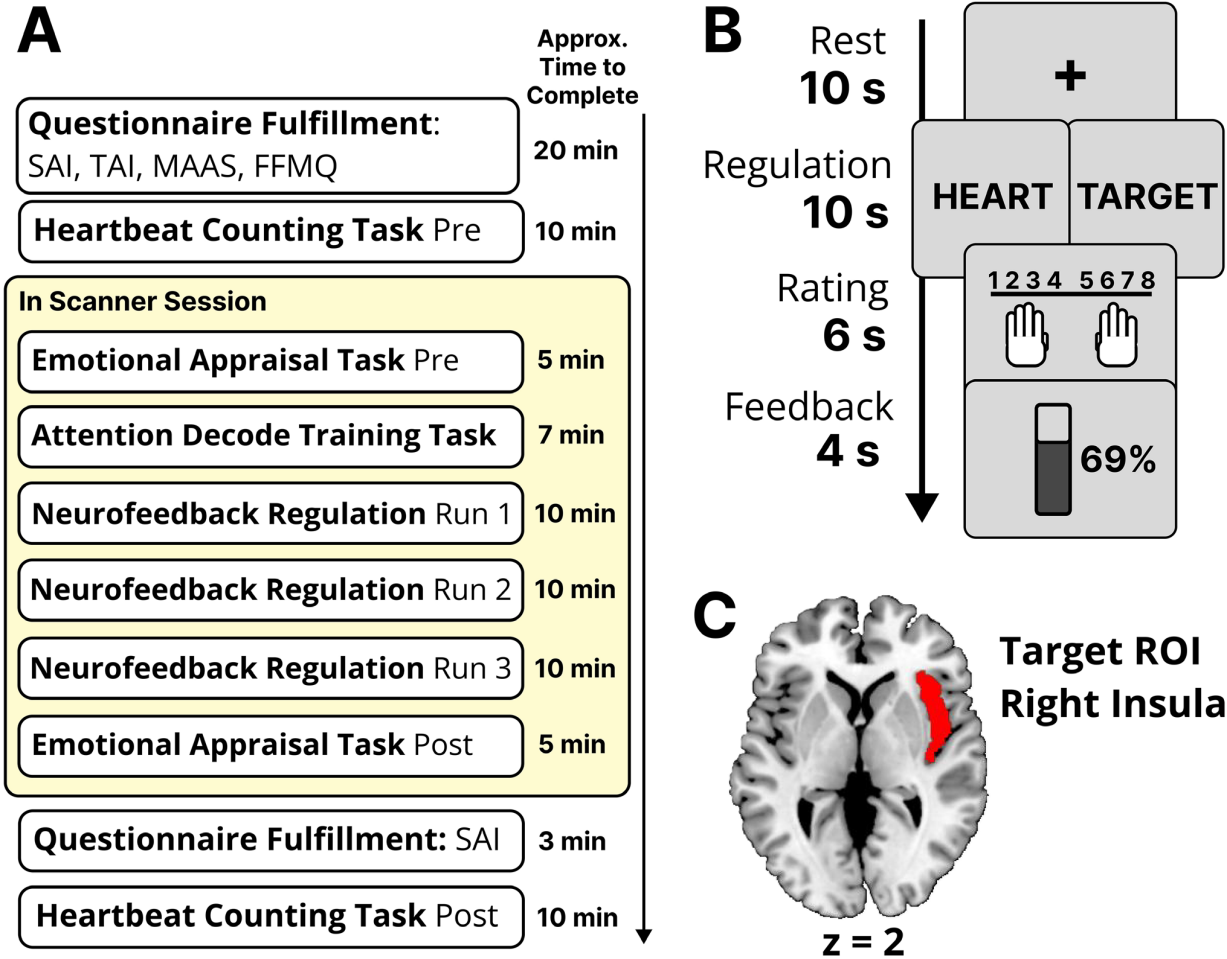
Experimental Design and Neurofeedback Training Protocol. (A) The experiment consisted of a single MRI scanning session, preceded and followed by behavioural tasks outside the scanner. Participants first completed self-report questionnaires, including the State Anxiety Inventory (SAI), Trait Anxiety Inventory (TAI), Five Facet Mindfulness Questionnaire (FFMQ), and Mindful Attention Awareness Scale (MAAS), followed by a heartbeat counting task. During the fMRI session, they completed an emotional appraisal task, an attention decoder training task, and three neurofeedback (NF) training runs. The session concluded with a post-training emotional appraisal task, a second heartbeat counting task, and a final SAI questionnaire. (B) Structure of a single NF trial. Each trial included a 10-second rest period, a 10-second regulation period (heartbeat or visual focus), a 6-second rating phase, and a 4-second feedback display. Feedback was presented intermittently as a numerical NF score and a graphical thermometer to enhance learning efficiency. (C) The right insula (red) was the region of interest (ROI) for NF training, defined using the Automated Anatomical Labeling (AAL) atlas. Real-time signal processing employed a linear support vector machine (SVM) to classify interoceptive and exteroceptive attention states.

As shown in Figure 1A, our single-session experiment consisted of an MRI scanning session, preceded and followed by behavioural tasks performed outside the scanner, with questionnaire completion. Upon providing informed consent, participants first completed a battery of self-report questionnaires in the following order: State Anxiety Inventory (SAI), Trait Anxiety Inventory (TAI) (Spielberger et al., 1970), Five Facet Mindfulness Questionnaire (FFMQ) (Baer et al., 2006), and Mindful Attention Awareness Scale (MAAS) (Brown & Ryan, 2003). The TAI, FFMQ, and MAAS were administered to assess individual differences in trait-level factors potentially influencing NF training outcomes, while the SAI was used to capture state-level anxiety. Validated Japanese versions of these questionnaires were administered (Fujino et al., 2015; Shimizu & Imasakae, 1981; Sugiura et al., 2012), and the item order was randomised for each participant to reduce response biases.

Next, participants performed a modified heartbeat counting task (Desmedt et al., 2020) to establish a baseline measure of cardiac interoceptive accuracy and subjective confidence about their task performance before NF training. Subsequently, they were briefed on the fMRI procedures. The in-scanner protocol comprised six runs in the following order: (1) a pre-training emotion appraisal task, (2) an interoceptive–exteroceptive attention decoder training task, (3) three consecutive NF regulation training runs, and (4) a post-training emotion appraisal task. After the fMRI session, participants again completed the SAI and repeated the heartbeat counting task to assess any post-training changes.

### 2.3 Questionnaires

The MAAS is a 15-item scale measuring dispositional mindfulness, or the ability to maintain awareness of and attention to present-moment experiences. The FFMQ assesses five facets of mindfulness—observing, describing, acting with awareness, non-judging of inner experience, and non-reactivity to inner experience—through 39 items. The SAI, which measures temporary, situational anxiety, and the TAI, which evaluates more stable, enduring anxiety levels. Each subscale contains 20 items, with higher scores indicating greater anxiety. The MAAS, FFMQ, and TAI were administered before the fMRI tasks to characterise trait-like dimensions, while the SAI was used before and after the NF training to assess how participants’ state anxiety might fluctuate.

### 2.4 Heartbeat counting task

Cardiac interoceptive accuracy was assessed before and after the NF training session using a modified heartbeat counting task. Participants were instructed to silently count their perceived heartbeats over varying durations (15, 25, 35, 45, 55, or 65 seconds) by focusing solely on internal bodily sensations. A pulse oximeter (Tokyo Devices) was used to monitor photoplethysmography signals throughout the task for an objective record of actual heartbeats. Participants were explicitly directed to avoid strategies unrelated to direct heartbeat perception, such as timing estimations or feeling pulses at peripheral sites (e.g., wrist, fingertip). This strict instruction has been shown to mitigate an inflation of interoceptive accuracy score (Desmedt et al., 2020). Six trials were administered (one trial per each duration), with the start and stop signals indicated by an auditory beep. Immediately after each trial, participants verbally reported the number of heartbeats perceived and rated their subjective confidence in this count on a scale ranging from 0 (no perception) to 100 (full perception). Interoceptive accuracy scores were then calculated for each trial using a standardised formula that compares the perceived number of heartbeats with the objectively recorded count, with the final score obtained by averaging across six trials as follows:

$$Interoceptive\;Accuracy=\frac{1}{6}\sum_{i\;=\;1}^{6}\left(1-\frac{\left|Recorded\;Nbeats_{i}\;-\;Reported\;Nbeats_{i}\right|}{Recorded\;Nbeats_{i}}\right).$$

This task is well suited for evaluating the effects of NF training on interoceptive accuracy because prior work has shown that heartbeat perception performance remains relatively stable across repeated assessments (Ferentzi et al., 2018; Santos et al., 2023) but can improve following targeted interventions using long-term contemplative training or auditory feedback of each heartbeat arrival (Bornemann & Singer, 2017; Meyerholz et al., 2019). Here, however, no feedback on heartbeat counts or interoceptive accuracy scores was provided, ensuring that any improvement would derive from the NF session rather than direct practice on heartbeat detection. By comparing pre- and post-training performance, we aimed to investigate whether regulating insular activation via NF could facilitate more accurate cardiac interoceptive awareness, potentially reflecting an enhanced capacity to focus on internal bodily signals.

### 2.5 MRI acquisition

MRI data were acquired at Hokkaido University on a 3-Tesla Siemens Prisma scanner (Erlangen, Germany) equipped with a 64-channel head coil. For functional imaging, T2*-weighted echo-planar imaging (EPI) was employed. A total of 202 volumes were collected during the decoder training run, and 298 volumes per NF run, each using a gradient EPI sequence. The first three volumes of each run were discarded to allow for T1 signal equilibration. The primary EPI parameters were TR = 2,000 ms, TE = 30 ms, flip angle = 90°, field of view = 192 × 192 mm, image matrix = 94 × 94, 35 axial slices, slice thickness = 3.0 mm, and inter-slice gap = 0.75 mm. Additionally, high-resolution T1-weighted anatomical images were acquired using an MP-RAGE sequence (TR = 2,300 ms, TE = 2.41 ms, flip angle = 8°, field of view = 256 × 256 mm, matrix = 256 × 256, 224 axial slices, and slice thickness = 0.8 mm with no gap) for spatial normalisation and coregistration in subsequent analyses.

### 2.6 Emotional appraisal task

To evaluate neural and behavioural responses to negatively valenced stimuli before and after NF training, participants performed an emotional appraisal task inside the MRI scanner. During each trial, they viewed an image and rated its valence on a Likert scale from 1 (extremely negative) to 8 (extremely positive) using a response pad. Stimuli were selected from the Open Affective Standardized Image Set dataset and comprised 18 negative and 18 neutral images (Kurdi et al., 2017). To ensure a balanced selection, images were selected equally as possible across four source categories (objects, scenes, animals, and people), with no more than three images selected from a single theme (e.g., acorn, beach, cat, doctor). For ethical reasons, we excluded images depicting highly distressing themes, such as dead bodies or nude women. Two equivalent sets—each containing nine negative and nine neutral stimuli—were prepared for the pre- and post-training sessions, with the order of presentation counterbalanced across participants to control for order and familiarity effects. Both image sets were carefully matched for mean valence and arousal ratings, accounting for gender differences to ensure comparability between sessions. Mean valence and arousal ratings, as assessed in the original study using a 7-point Likert scale, were as follows: Negative Set 1: valence = 2.131 ± 0.442, arousal = 4.171 ± 0.763; Negative Set 2: valence = 2.261 ± 0.363, arousal = 4.083 ± 0.615; Neutral Set 1: valence = 4.077 ± 0.151, arousal = 3.141 ± 0.676; Neutral Set 2: valence = 4.065 ± 0.208, arousal = 2.935 ± 0.868.

This task was included to investigate whether regulating insular activation through NF would modulate participants’ emotional reactivity to negative stimuli, both at the subjective (valence ratings) and neural (fMRI) levels. Previous work has shown that individuals with mindfulness experience often exhibit diminished neural and physiological responses to negative emotional stimuli (e.g., in amygdala activation), suggesting a possible link between heightened interoceptive awareness and reduced emotional reactivity (Farb et al., 2010). By comparing emotional ratings and neural activations in affective processing regions—including the amygdala and prefrontal cortices—before and after NF training, we aimed to determine whether improving interoceptive attention via insula-based NF could yield patterns similar to those observed in mindfulness practitioners.

### 2.7 Interoceptive–exteroceptive attention decoder training task

To establish reliable insular activation patterns for both interoceptive and exteroceptive attention, we employed an attention decoder training task before the NF runs. This task aimed to collect individual-specific patterns of insular activity during heartbeat-focused (interoceptive) and visually focused (exteroceptive) attention, which were then used to train a support vector machine (SVM) decoder. Such a decoder allows for real-time classification between the two attentional states in subsequent NF training. A total of 24 trials were administered: alternating 12 heartbeat-attention trials and 12 visual-attention trials, each lasting 10 seconds and interleaved with 12-second rest periods. This design followed prior work indicating that short blocks of directed internal versus external focus reliably engage the insula in a state-specific manner (Kerr et al., 2016; Simmons et al., 2013; Wiebking et al., 2010).

During heartbeat-attention trials, the word “HEART” was presented in black at the centre of the screen, while during visual-attention trials, the word “TARGET” appeared instead. These distinct cue words allowed participants to clearly recognise which condition they were in and engage accordingly. In the visual-attention condition, the blackness of “TARGET” subtly decreased on each volume, creating gradual greyscale changes. Participants were instructed to concentrate on perceiving their own heartbeats without resorting to timing cues or palpating peripheral pulses in the heartbeat-attention condition. By contrast, during visual-attention trials, they were asked to track subtle greyscale changes in a word displayed on the screen. The use of greyscale shifts, rather than obvious colour changes, served to mimic the subtlety of interoceptive signals and minimise abrupt external distractions as in a previous study (Haruki & Ogawa, 2023). Data from these two conditions were then utilised to train the SVM to classify voxel-wise activation patterns in the right insula, thereby providing the foundation for the NF score calculation in the subsequent regulation runs. We chose to train the decoder on two active attentional states for both conceptual and technical reasons, in addition to prior findings that decoding-based neurofeedback can effectively train sustained attention by enhancing the neural discriminability of attentional states (deBettencourt et al., 2015). Conceptually, alternating between an interoceptive and easier exteroceptive task provides a clear contrast that facilitates learning for participants naive to sustained interoceptive focus, as shown in previous studies (Haruki & Ogawa, 2023; Simmons et al., 2013). This “attentional switching” design is analogous to established interventions such as body scan meditation, which aim to train flexible attentional shifts to improve body awareness (Bornemann & Singer, 2017; Fischer et al., 2017). Technically, this design provides two well-balanced conditions for training a meaningful SVM decoder. A simpler contrast, such as interoceptive attention versus rest, could risk a ceiling effect in classification accuracy, as the neural signatures for active tasks versus rest are often highly separable and thus provide less informative gradients for learning fine-grained neural control.

### 2.8 Neurofeedback regulation training

Participants underwent three regulation runs, each designed to help them modulate insular activation patterns in real time. Each run began with a 20-second fixation (baseline), followed by 24 alternating blocks (12 interoceptive and 12 exteroceptive), mirroring the setup from the interoceptive–exteroceptive attention decoder training task. Within each block, participants engaged in (i) a 10-second rest interval; (ii) 10-second “regulation” period to focus solely on either their heartbeat (interoceptive) or subtle greyscale changes in a word colour (exteroceptive); (iii) a 6-second rating period, during which they reported the perceived intensity of the targeted sensation (1 = not at all intense, 8 = extremely intense); and (iv) a 4-second feedback display showing a numeric NF score alongside a graphical thermometer (Fig. 1B). During the 10-second regulation period within each block, the word “HEART” (for interoceptive) or “TARGET” (for exteroceptive) was displayed at the centre of the screen to indicate the current condition. Unlike a previous study using continuous feedback updated every TR (Zhang et al., 2023), we employed intermittent feedback, presenting a single feedback update per block. This approach was guided by evidence that intermittent feedback reduces cognitive load, facilitates strategy consolidation, and enhances learning efficiency (Hellrung et al., 2018; Johnson et al., 2012). Moreover, because continuous feedback demands sustained attention to external cues, it may disrupt the interoceptive–exteroceptive shifts central to our design. Intermittent feedback, by contrast, is better suited for single-session NF paradigms where minimising distraction while promoting effective self-regulation is essential (Emmert, Kopel, et al., 2017).

Real-time image preprocessing was carried out by an in-house software using modules of SPM8 (Wellcome Department of Cognitive Neurology). To ensure volume-to-volume spatial alignment across the scan and account for head motion, a real-time realignment procedure was implemented. For each run, the first functional volume was taken as a reference image, and every subsequent volume was co-registered in real-time to this initial reference. This ensured that the region of interest (ROI) mask was applied to a consistently aligned anatomical space throughout the training. The ROI was defined by using the right insula from the Automated Anatomical Labeling (AAL) atlas (Fig. 1C). Voxel-wise percent signal change (PSC) within the ROI were then computed relative to the initial baseline period (20 seconds). Our decision to employ a pattern-based decoder rather than providing feedback on the average BOLD signal of the ROI was based on the unique nature of the interoceptive attention task. This task requires a fragile internal focus that is highly susceptible to disruption by external stimuli (Haruki & Ogawa, 2024). This concern was substantiated by our own unpublished pilot data (2019; N = 15), in which standard continuous feedback of the functionally localised ROI-average signal paradoxically decreased insula activity during interoceptive focus, deviating from the results of Zhang et al. (2023). Therefore, to provide a more functionally specific feedback signal, we employed a linear SVM (LIBSVM, http://www.csie.ntu.edu.tw/~cjlin/libsvm) with a regularisation parameter C = 1 to classify whether the participant’s activation pattern was more similar to the interoceptive (heartbeat-focused) or exteroceptive (visual-focused) attention condition.

**Fig. 2.**
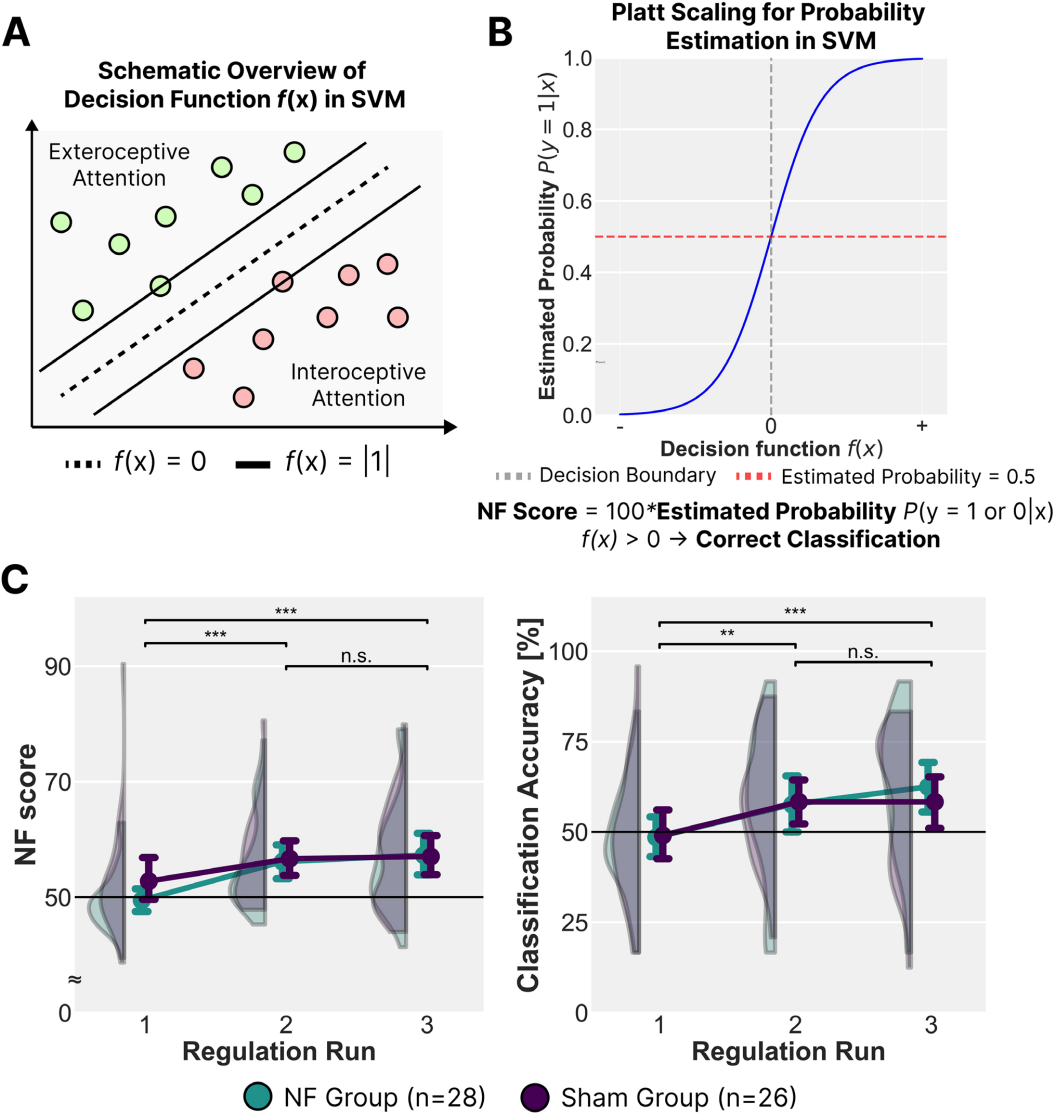
Neurofeedback Classification and Training Performance for Each Group. (A) A support vector machine (SVM) decoder was trained to distinguish interoceptive (heartbeat-focused) and exteroceptive (visual-focused) attention states based on four-dimensional fMRI data from the right insula. The decision function *f*(x) determined classification boundaries between these states. (B) The neurofeedback (NF) score was computed using Platt scaling, which transformed the SVM decision function into a probability estimate. During interoceptive attention blocks, a higher NF score indicated stronger classification as the interoceptive attention state, while during exteroceptive attention blocks, the NF score was calculated as 100 minus the probability, ensuring that higher values consistently reflected better classification performance. (C) NF scores and classification accuracy (CA) across three regulation runs for the NF group (n = 28) and Sham group (n = 26). Both measures increased over runs, indicating improved separation of brain activity patterns, though no significant differences between groups were observed. Error bars indicate mean ± SEM. Asterisks denote a significant increase from Run 1 to Run 2, based on post hoc t-tests with Bonferroni correction (**: *p* < .01, ***: *p* < .001). n.s. indicates not significant (*p* > .05).

The decoder was trained to find an optimal decision function that separated the two states (Fig. 2A). The actual input labelled as interoceptive or exteroceptive states to the decoder consisted of four-dimensional fMRI data, integrating both spatial and temporal features of right insula activation. The spatial component was represented by voxel-wise percent signal change within the ROI, which was not normalised, preserving the amplitude information of each voxel. The temporal component included activation values from the last five volumes within each block. This approach ensured that the SVM decoder could effectively distinguish between interoceptive and exteroceptive states by utilising the spatiotemporal dynamics during the preceding regulation trial.

The NF score was computed as a probability value derived from the SVM decision function using Platt scaling (Fig. 2B). Specifically, the SVM decoder’s raw decision value, which represents the signed distance from the decision boundary, was transformed into a probability estimate using the following logistic function:

$$P\left(y=1|x\right)=\frac{1}{1+exp\left(A_{r}f\left(x\right)+B_{r}\right)}$$,

where *A_r_* and *B_r_* are scaling parameters that were re-estimated at each regulation run *r*. These parameters were obtained by fitting the logistic function to the SVM’s decision values using data from both the attention decoder training task and prior runs. The NF score was then defined as

NF Score=100×P(y=1|x),

where higher values indicated stronger classification as the interoceptive attention state, while lower values indicated stronger classification as the exteroceptive attention state. During visual attention blocks, the NF score was instead computed as

NF Score=100×(1−P(y=1|x)),

ensuring that feedback always reflected the decoder’s confidence in the instructed attentional state. If the NF score exceeded 50, the trial was considered correctly classified, meaning the SVM decoder successfully followed the instructed attentional state (interoceptive or exteroceptive) for that block. In Run 1, the decoder was trained exclusively on data from the preceding attention decoder training task. In Runs 2 and 3, it was incrementally retrained, incorporating data from both previous regulation runs and the original decoder training task, progressively refining its ability to distinguish interoceptive from exteroceptive attention states. This adaptive learning strategy ensured that the probability transformation remained calibrated across runs, improving classification stability and feedback interpretability (Abdennour et al., 2025).

In the Sham group, participants did not receive real-time NF scores derived from their brain activity. Instead, they were presented with a Sham score, which was statistically matched to the NF scores observed in the NF group (Misaki et al., 2024; Tsuchiyagaito et al., 2023). The Sham scores were generated based on the data from the initial 26 participants in the NF group, ensuring that the statistical properties of the displayed feedback were indistinguishable from real NF scores. Specifically, for each participant in the Sham group, the initial Sham score was sampled from the unconditional probability distribution *P*(*score*) of NF scores, preserving the overall score distribution observed in the NF group. In each trial, the success or failure of the trial was determined probabilistically based on the run-specific correct classification rate from the NF group (Run 1 = 48.512, Run 2 = 58.589, Run 3 = 62.500). If a trial was categorized as successful, the Sham score was sampled from a range corresponding to correctly classified trials (*score* > 50); if it was categorized as a failure, the score was sampled from incorrectly classified trials (*score* ≤ 50). To ensure temporal consistency, the next Sham score *score*_t+1_ was generated based on the conditional probability distribution *P*(*score*_t+1_|*score_t_*) of transitions between score categories, derived from the NF group data. If no transition probability was observed for a given score category, the next score was sampled from the unconditional probability distribution *P*(*score*). This method ensured that the Sham group experienced feedback patterns that mimicked the variability and statistical structure of real NF scores, while preventing any meaningful brain-state-dependent learning.

Participants were given clear instructions on how to interpret the NF score. They were informed that a score of 50 indicated an ambiguous classification, meaning their brain activity pattern cannot be identified as either the interoceptive or exteroceptive attention state. A score above 50 suggested successful alignment with the instructed attentional focus, while a lower score indicated a closer match to the opposite state. Participants were encouraged to focus on their assigned task—either heartbeat perception or detecting subtle colour changes—and to use the NF score after each trial to assess whether they were effectively modulating their attentional focus thereby the brain activation. Additionally, they were informed that the 6-second rating period served two purposes. First, it compensated for the haemodynamic delay, ensuring that the NF score accurately reflected brain activity from the preceding regulation period. Second, it maintained engagement by prompting participants to evaluate the subjective intensity of their experience. This design provided a structured and interpretable measure of how distinctly their right insula activity differentiated interoceptive from exteroceptive attention, potentially enhancing their ability to voluntarily regulate interoceptive focus. However, due to a technical script error during data acquisition, these block-by-block intensity ratings were not saved for a substantial portion of the sample, precluding a formal analysis of these data.

### 2.9 Offline fMRI analysis

We investigated the neural activation during the NF regulation training and emotional appraisal task, separately. Before the statistical inferences, image preprocessing was performed using SPM12 software (Wellcome Department of Cognitive Neurology; http://www.fil.ion.ucl.ac.uk/spm). All the functional images were initially realigned to adjust for motion-related artefacts. Volume-based realignment was performed by co-registering images using rigid-body transformation to minimise the squared differences between volumes. The realigned images were then spatially normalised with the Montreal Neurologic Institute template based on the affine and nonlinear registration of co-registered T1-weighted anatomic images. They were resampled into 3 mm-cube voxels with sinc interpolation. The images were spatially smoothed using a Gaussian kernel of 6 x 6 x 6 mm full-width at half-maximum.

For NF regulation training, an individual-level GLM was specified to capture condition-specific activity across the three runs for each participant. Two regressors of interest—one for heartbeat-attention blocks and one for visual-attention blocks—were modelled as boxcar functions (duration = 10 seconds each) convolved with the canonical haemodynamic response function (HRF). Rest, rating, and feedback periods were not modelled and used as the baseline. Six motion parameters (three translation, three rotation) were included as nuisance regressors to partial out residual movement effects. From these individual models, we generated contrast images comparing heartbeat > visual and visual > heartbeat for each participant. At the group level, we conducted a random-effects analysis to (1) examine the overall activation pattern across all participants for each contrast (using one-sample t-tests) and (2) directly compare the NF and Sham groups (using two-sample t-tests). Significance was assessed using a voxel-wise threshold of *p* < .001 (uncorrected) with an extent threshold of *p* < .05, corrected for family-wise error (FWE). This approach allowed us to identify regions showing consistent differences in activation between the interoceptive (heartbeat) and exteroceptive (visual) focus conditions, as well as potential group-by-condition interactions attributable to the real-time NF.

Subsequently, we conducted an ROI analysis focusing on the feedback target, the right insula. First, we examined whether mean activation within this ROI differed between groups. This analysis was motivated by two considerations. Zhang et al. (2023) reported that participants receiving contingent feedback exhibited greater activation in their functionally localised feedback ROI (left anterior insula) compared with a Sham control group, suggesting a potential group difference in overall activation levels. Furthermore, given that raw PSC values served as input to the SVM decoder during the real-time analysis, it remains possible that classification performance was driven more by absolute activation differences between conditions rather than distinct multivariate patterns. To evaluate these possibilities, we extracted beta estimates from the right insula separately for heartbeat- and visual-attention conditions in each regulation run. We then conducted repeated-measures ANOVAs for each condition, with run (1, 2, and 3) as a within-subject factor and group (NF, Sham) as a between-subject factor.

Beyond univariate activation analysis, we performed an offline multivoxel pattern analysis (MVPA) to assess whether NF training modulated the distinctiveness of activation patterns for interoceptive and exteroceptive attention within the right insula. To obtain trial-specific features for this analysis while preserving fine-grained spatial information, we performed a separate first-level GLM on the unsmoothed functional data. From this GLM, a beta estimate was computed for each individual trial. Similar to the real-time analysis, a linear SVM decoder (C = 1) was trained to classify heartbeat versus visual attention states based on voxel-wise beta estimates. For each task run (24 trials per run), a separate beta was estimated for each trial, allowing multivoxel patterns to be extracted at the trial level. Trials within each run (24 per run) were split into two subsets, allowing offline classification accuracy to be estimated separately for the attention decoder training task and each of the three NF regulation runs using a two-fold cross-validation. Unlike the real-time approach, which relied on moment-to-moment signal fluctuations, this offline analysis used GLM-derived beta estimates computed for each individual trial, presumably providing temporally denoised and trial-specific activation patterns that offer improved signal stability. To formally assess whether NF training influenced the discriminability of interoceptive and exteroceptive attention states, we conducted a repeated-measures ANOVA on classification accuracy, with run (Training, Regulation 1, 2, 3) as a within-subject factor and group (NF, Sham) as a between-subject factor. This allowed us to determine whether changes in representational separation emerged over the course of NF training and whether these changes differed between groups. While both the online NF score and this offline MVPA index the separability of interoceptive and exteroceptive attention states, they differ fundamentally in their computational pipelines: the online score reflects transient, moment-to-moment classification performance within the feedback loop, whereas the offline analysis quantifies post hoc separability of trial-averaged, denoised activation patterns, which yields predictably different outputs (Heunis et al., 2020; Ramot & Gonzalez-Castillo, 2019).

We analysed brain responses during the emotional appraisal task using a two-level framework. At the individual level, two regressors were specified: negative-image trials and neutral-image trials, each modelled as a boxcar function convolved with the canonical HRF. We next focused on an ROI analysis in the bilateral amygdala and insula, given its established role in emotional processing and its potential modulation by attentional or mindfulness-based interventions (Doll et al., 2016; Kral et al., 2018; Lutz et al., 2014). The ROIs were defined using the AAL atlas, and mean beta coefficients for each valence were extracted separately for the bilateral amygdala and insula in each participant. These beta values were subjected to repeated-measures ANOVAs, with session (pre-, post-NF training) and valence (neutral, negative) as within-subject factors, and group (NF, Sham) as a between-subject factor. One participant from the Sham group was excluded from these analyses due to a lack of behavioural responses in eight trials during the post-NF session. This analytic approach aimed to assess whether targeting the insula through NF training might indirectly influence reactivity in emotion-related regions, as indicated by changes in these ROIs response to negative stimuli from pre- to post-NF training.

## 3 Results

Descriptive data for each group are presented in Table 1.

**Table 1.** Descriptive statistics of demographic variables and questionnaire measures between groups.

	Count NF Group	Count Sham Group	Group Difference *χ*^2^_1_	*p*			
Men	16	16	0.108	.743			
Women	12	10					

	Mean NF Group	Mean Sham Group	Group Difference *t*_52_	Uncorrected *p*	Cohen’s *d*	Correlation with LE *r*	Uncorrected *p*
Age	24.286 ± 2.566	21.692 ± 2.035	4.094	>.001	1.115	-.152	.274
TAI	48.643 ± 10.012	46.077 ± 10.518	0.918	.363	0.250	.060	.665
MAAS	48.714 ± 12.180	58.423 ± 12.245	-2.919	.005	-0.795	-.238	.083
SAI-Pre	37.893 ± 6.641	37.808 ± 8.139	0.042	.966	0.012	.241	.079
SAI-Post	38.821 ± 7.232	39.769 ± 11.769	-0.356	.723	-0.097	-.041	.766
FFMQ							
Observing	24.893 ± 4.864	25.423 ± 4.263	-0.425	.673	-0.116	.096	.488
Nonreactivity	16.143 ± 4.284	17.269 ± 4.729	-0.918	.363	-0.250	-.001	.995
Nonjudging	23.429 ± 5.922	25.654 ± 7.499	-1.215	.230	-0.331	.040	.773
Describing	23.464 ± 7.351	23.615 ± 6.585	-0.079	.937	-0.022	.036	.798
Acting with awareness	23.500 ± 5.872	25.154 ± 6.798	-0.959	.342	-0.261	-.126	.365

Group-wise comparisons of demographic variables and questionnaire measures between the neurofeedback (NF) group (n = 28) and the Sham group (n = 26). The table includes mean values (±SD), group differences tested using chi-square tests for categorical variables, and t-tests for continuous variables, uncorrected *p*-values, effect sizes (Cohen’s *d*), and Pearson’s correlation coefficients (*r*) with the NF learning effect (LE). Questionnaire measures include the Trait Anxiety Inventory (TAI), State Anxiety Inventory (SAI) before and after NF training (SAI-Pre, SAI-Post), Mindful Attention Awareness Scale (MAAS), and Five Facet Mindfulness Questionnaire (FFMQ) subscales (Observing, Nonreactivity, Nonjudging, Describing, Acting with Awareness).

### 3.1 Group-level regulation results of the NF training

We first evaluated the effectiveness of the NF training by examining two measures of regulation performance recorded in each run: the mean NF score, which is the average SVM decoder-derived probability for each trial, and classification accuracy (CA), which is the proportion of trials correctly classified (heartbeat vs. visual) out of 24. A significant learning effect was observed in the NF group for both NF score and CA, whereas no such effect was found in the Sham group; however, direct comparisons between groups did not reveal statistically significant differences, as detailed below.

Repeated-measures ANOVAs were conducted with run (1, 2, 3) as a within-subject factor and group (NF, Sham) as a between-subject factor. These analyses revealed no significant main effect of group (NF score: *F*_1, 52_ = 0.428, *η*^2^_p_ = .008, *p* = .516; CA: *F*_1, 52_ = 0.070, *η*^2^_p_ = .001, *p* = .792) or group–run interaction (NF score: *F*_2, 104_ = 1.189, *η*^2^_p_ = .022, *p* = .309; CA: *F*_2, 104_ = 0.444, *η*^2^_p_ = .008, *p* = .634). However, there was a notable main effect of run for both the NF score (*F*_2, 104_ = 14.876, *η*^2^_p_ = .222, *p* < .001) and CA (*F*_2, 104_ = 8.685, *η*^2^_p_ = .143, *p* < .001), indicating a sharp increase in performance between Run 1 and Run 2 (NF score: *t* = 4.385, *p* < .001; CA: *t* = 3.119, *p* = .007, with Bonferroni correction) (Fig. 2C). This abrupt improvement was likely influenced by the SVM update procedure, which may have obscured incremental differences between groups. Nonetheless, we observed that the simple main effect of run was stronger in the NF group (*F* = 13.020, *p* < .001) than in the Sham group (*F* = 3.606, *p* = .034), tentatively suggesting better regulation at a group level when contingent feedback was provided.

To further clarify this pattern, we computed a learning effect that captured improvements from the earlier two runs to the final run as

Learning Effect NF score and CA   =Run 3−12(Run 1+Run 2).

**Fig. 3.**
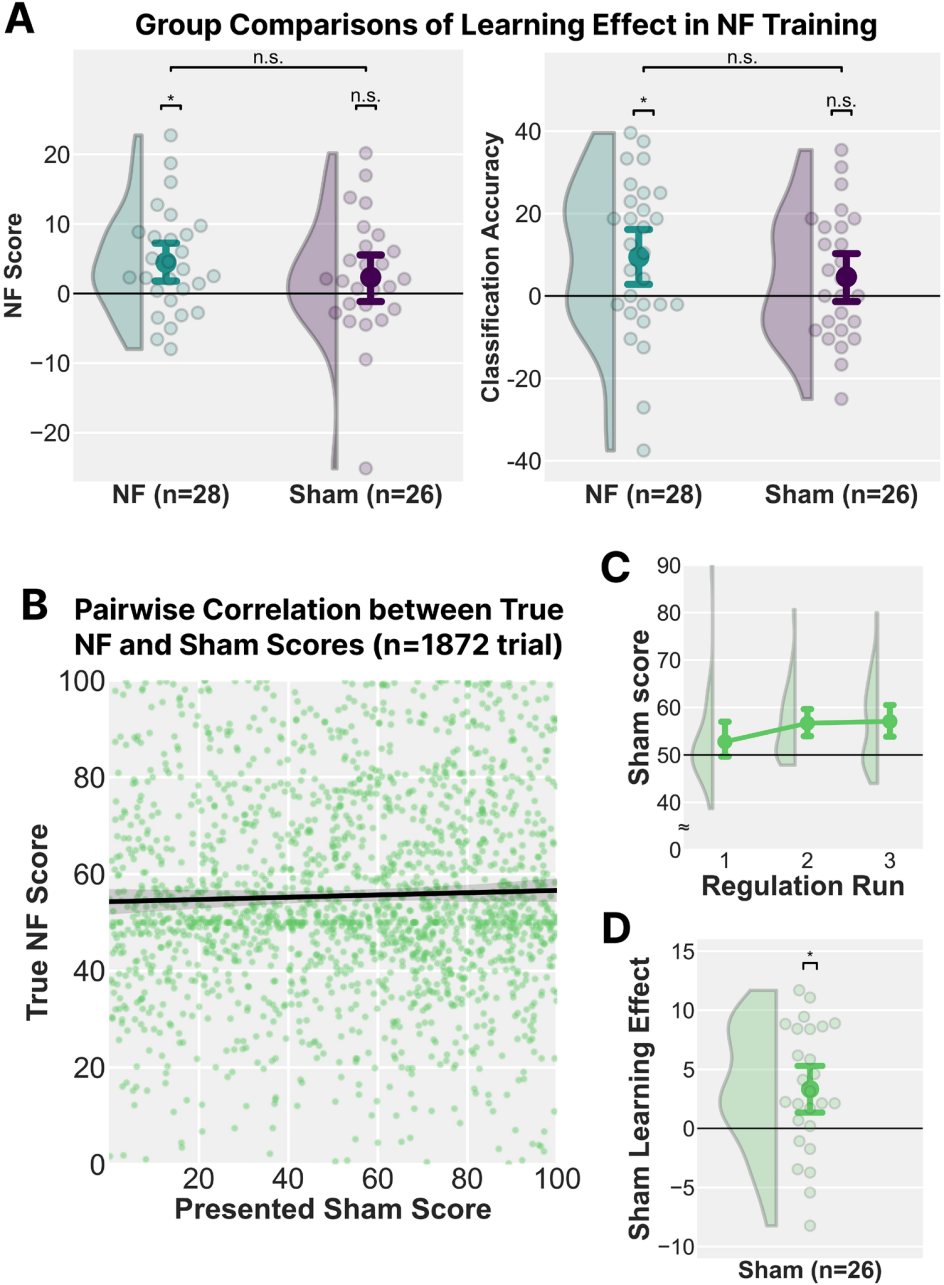
Evaluation of Learning Effects and Sham Feedback. (A) Learning effects in neurofeedback (NF) training for each group. One-sample t-tests confirmed the learning effect was significantly greater than zero for the NF group (n = 28) in both NF score and classification accuracy (CA), while no such effect was observed in the Sham group (n = 26). However, the group difference did not reach significance. (B) Pairwise correlation between the presented sham scores and the true (but unobservable to the participants) NF scores in the corresponding trials (n = 1,872 trials). No correlation was observed (BF_10_ = 0.078), confirming that the sham feedback was unrelated to participants’ actual brain activity. (C) Mean sham scores across regulation runs in the Sham group (n = 26). Scores were statistically matched to mimic the distribution of the NF group. (D) Apparent learning effect in the Sham group based on presented sham scores. Despite an increasing trend in sham feedback, this did not reflect actual insula-based regulation. Error bars indicate mean ± SEM. *: *p* < .05; n.s.: *p* > .05.

Because performance showed a sharp increase between Run 1 and Run 2, a simple comparison between Run 1 and Run 3 may not accurately reflect gradual learning. We, therefore, used the average of Runs 1 and 2 as a baseline, which also helps to stabilise early-session variability due to familiarisation and strategy adjustment. One-sample t-tests showed that this learning effect was significantly greater than zero in the NF group for both the NF score (NF score: *t*_27_ = 3.161, Cohen’s *d* = 0.597, 95% confidence interval (CI) = [0.190, 0.996], *p* = .004) and CA (*t*_27_ = 2.629, *p* = .014, *d* = 0.497, 95% CI = [0.100, 0.886], *p* = .004), whereas no such effect emerged in the Sham group (NF score: *t*_25_ = 1.326, *d* = 0.260, 95% CI = [-0.133, 0.649], *p* = .197; CA: *t*_25_ = 1.524, *d* = 0.299, 95% CI = [-0.097, 0.689], *p* = .140) (Fig. 3A). Direct between-group comparisons on these learning-effect metrics, however, were not statistically significant (NF score: *t*_52_ = 0.948, *d* = 0.258, 95% CI = [-0.279, 0.793], *p* = .348; CA: *t*_52_ = 1.011, *d* = 0.275, 95% CI = [-0.262, 0.810], *p* = .317). To further probe the specificity of this learning, we conducted a post hoc analysis separating the learning effect by trial type. Within the NF group, a significant learning effect was observed specifically for interoceptive attention trials in both the NF score (Group Mean = 8.150 ± 14.203; *t*_27_ = 3.036, *p* = .005, *d* = 0.575) and CA (M = 0.156 ± 0.280; *t*_27_ = 2.958, *p* = .006, *d* = 0.559). In contrast, the learning effect in their exteroceptive trials was not significant for either the NF score (M = 0.769 ± 11.904; *t*_27_ = 0.342, *p* = .735, *d* = 0.065) or CA (M = 0.033 ± 0.351; *t*_27_ = 0.494, *p* = .626, *d* = 0.093). Crucially, this pattern was absent in the Sham group, who showed no significant learning effect in any condition: interoceptive attention trials (NF score: M = 3.472 ± 17.318, *t*_25_ = 1.022, *p* = .316, *d* = 0.201; CA: M = 0.090 ± 0.354, *t*_25_ = 1.295, *p* = .207, *d* = 0.254) or exteroceptive trials (NF score: M = 1.198 ± 19.631, *t*_25_ = 0.311, *p* = .758, *d* = 0.061; CA: M = 0.003 ± 0.358, *t*_25_ = 0.046, *p* = .964, *d* = 0.009). While a direct between-group comparison of the interoceptive-specific learning effect did not reach significance, the overall pattern of results strongly suggests that the NF training selectively enhanced the ability to regulate insula activity related to interoceptive attention.

To clarify for the purpose of statistical comparison, the “true” NF scores for the Sham group were calculated from their brain activity in the same manner as the NF group but were not displayed to the participants. Participants in the Sham group did not receive these true NF scores but instead saw “sham” scores that were statistically matched to the NF group. Pairwise correlation between the sham score and true (but unobservable to the participants) NF score in the corresponding trial provided strong evidence for no correlation (Pearson’s *rho* = .033, 95% credible interval = [-0.013, 0.078], BF_10_ = 0.078) (Fig. 3B). The mean sham scores across individuals were 50.850 ± 4.242 in Run 1, 54.839 ± 5.424 in Run 2, and 56.174 ± 4.157 in Run 3 (Fig. 3C). Moreover, the sham scores displayed to participants exhibited an apparent improvement across runs; a one-sample t-test on the learning effect (Run 3 - ½(Run 1 + Run 2) confirmed this increase was significantly greater than zero (*t*_25_ = 3.204, *p* = .004, *d* = 0.628, 95% CI = [0.202, 1.045]; Fig. 3D), suggesting that the sham feedback successfully mimicked performance improvement without reflecting true insula-based regulation. That is, although participants in the Sham group saw ostensibly improving sham feedback, it did not translate into true insula-based regulation learning (see Fig. 3A Sham).

Moreover, we explored individual factors associated with successful regulation by examining correlations between the learning effect of the NF score, age, and questionnaire measures, including each FFMQ factor, total MAAS score, pre- and post-NF SAI measures, and TAI score. However, no significant correlations were observed (Pearson’s |*r*|s < .170, uncorrected-*p*’s > .387) (for full statistics, see Table 1). These results suggest that the learning effect observed in NF training may not be directly explained by general mindfulness, attention, or anxiety traits as assessed by these measures.

### 3.2 Brain activation during the NF regulation training

**Fig. 4.**
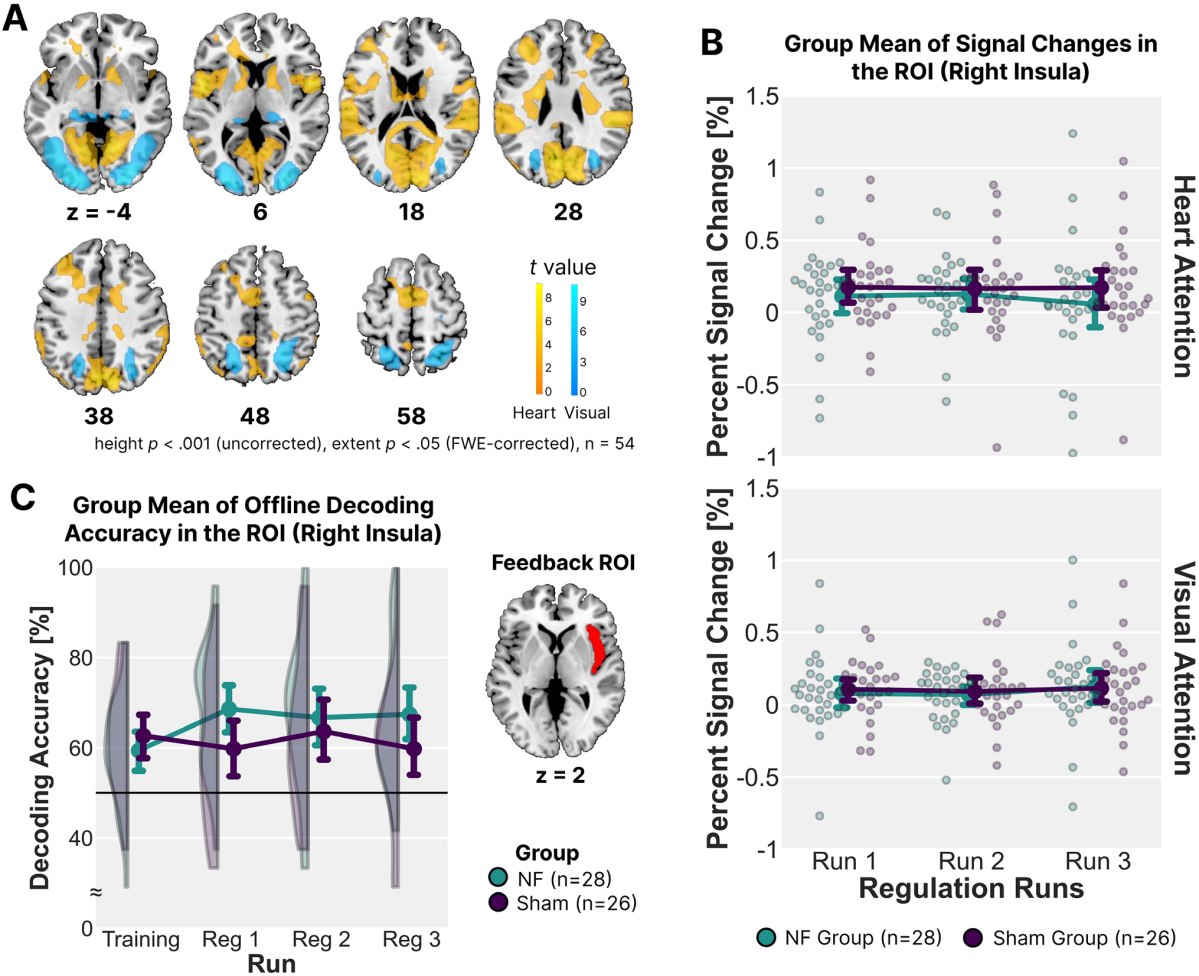
Whole-Brain and ROI Analyses of Neural Activation During Neurofeedback Training. (A) Whole-brain contrasts of heartbeat-focused versus visual-focused attention across all participants (n = 54). Regions showing greater activation for heartbeat attention (hot colour map) included the bilateral insula, frontal operculum, middle cingulate gyrus, and caudate, while regions with greater activation for visual attention (cold colour map) included the occipital cortex, superior parietal lobule, hippocampus, and cerebellum (voxel-level *p* < .001, cluster-level *p* < .05 FWE-corrected). (B) Group mean percent signal change in the right insula, the region of interest (ROI) used for neurofeedback (NF) training, across regulation runs. No significant differences were observed between the NF and Sham groups in either condition. (C) Offline classification accuracy of interoceptive and exteroceptive attention states within the right insula, calculated for the attentional decoder training task and each regulation run. A significant group × run interaction was observed, with the NF group showing improved classification accuracy in early runs compared with the decoder training task, while no such trend was seen in the Sham group. The right insula ROI was defined using the Automated Anatomical Labeling (AAL) atlas (red). Error bars indicate mean ± SEM.

To verify that our regulation task elicited the expected neural patterns for heartbeat- and visual-attention states, we performed a whole-brain analysis directly contrasting heartbeat > visual and visual > heartbeat at the group level. One-sample t-tests using data from all participants (voxel-level *p* < .001, cluster-level *p* < .05 FWE-corrected) revealed widespread activation in the heartbeat > visual contrast, including the bilateral middle insula, frontal operculum, middle cingulate gyrus, parietal operculum, caudate, dorsolateral prefrontal cortex, and cuneus (Fig. 4A; Table 2). Conversely, visual > heartbeat elicited significant activation in the bilateral occipital cortex, superior parietal cortex, hippocampus, and cerebellar vermis. These patterns are broadly consistent with previous studies (Haruki & Ogawa, 2023; Simmons et al., 2013), indicating that the current heartbeat/visual-attention paradigm—administered alongside real-time NF training—activated well-established interoceptive and exteroceptive attentional networks. Next, we compared these same contrasts between the NF and Sham groups to explore whether contingent feedback altered overall task-related activation. No cluster survived correction for multiple comparisons in either direction (NF > Sham or Sham > NF), suggesting that, despite the group-level differences in learning effects, the basic neural responses to heartbeat and visual-attention tasks remained largely similar across conditions.

**Table 2.** Brain activation during heartbeat and visual attention conditions.

Coordinates					
Region	x	y	z	T	Voxels in cluster
Heart vs. Visual					
L Cuneus	-6	-70	29	9.63	3064
R Cuneus	9	-70	29	8.02	
	12	-79	35	7.66	
L Frontal operculum	-51	2	5	8.73	3296
Caudate	-24	-4	29	6.89	
Superior temporal gyrus	-63	-28	20	6.89	
R Frontal operculum	51	2	8	7.21	290
Precentral gyrus	63	5	20	4.79	
Middle insula	36	8	8	4.70	
R Supramarginal gyrus	51	-34	29	7.10	512
Superior temporal gyrus	57	-28	20	6.56	
Angular gyrus	48	-64	44	5.70	
R Middle frontal gyrus	33	44	29	5.03	112
	27	35	29	4.00	
R Precentral gyrus	51	2	47	5.00	25
R Caudate	9	29	2	4.99	31
	21	35	-1	4.52	
R Temporal middle gyrus	57	-61	8	4.86	15
	54	-64	17	4.08	
R Middle cingulate	15	-28	41	4.73	43
					
Visual vs. heart					
L Middle occipital gyrus	-27	-94	-1	10.98	873
	-18	-97	-7	8.87	
	-45	-67	-7	7.67	
R Middle occipital gyrus	30	-91	2	10.87	1230
	24	-94	-4	9.48	
Superior parietal lobule	24	-58	53	7.47	
L Superior parietal lobule	-24	-61	50	7.41	414
	-24	-52	47	6.33	
Superior occipital gyrus	-27	-70	29	5.22	
L Cerebellum	-18	-40	-43	6.96	316
	-3	-73	-25	6.92	
	-6	-73	-34	6.91	
L Thalamus	-6	-31	-4	6.36	179
Hippocampus	-18	-34	2	5.42	
R Hippocampus	27	-28	-1	5.26	
R Cerebellum	18	-40	-43	5.43	45
	18	-55	-43	4.40	
Vermis	0	-55	-34	5.13	53
L Hippocampus	-27	-7	-16	4.61	17
R Hippocampus	30	-7	-16	4.34	18
R Precentral gyrus	27	-19	53	4.02	16

Whole-brain contrasts of heartbeat-focused attention versus visual-focused attention, and vice versa, during neurofeedback (NF) training runs across all participants (n = 54). The table lists significantly activated brain regions with their corresponding Montreal Neurological Institute (MNI) coordinates (x, y, z), peak *t*-values, and cluster sizes. Statistical thresholds were set at voxel-level *p* < .001 and cluster-level *p* < .05 family-wise error (FWE) corrected. L = left, R = right.

Following the whole-brain analysis, we conducted ROI analyses focusing on the feedback target, the right insula, to assess both overall activation levels and the pattern distinctiveness of interoceptive and exteroceptive attention. We first examined whether mean activation within the right insula differed between groups, by extracting beta estimates from the right insula separately for heartbeat and visual-attention conditions in each regulation run. We then conducted repeated measures ANOVAs for each condition, with session (Pre, Post) as a within-subject factor and group (NF, Sham) as a between-subject factor (Fig. 4B). Results indicated that in the heartbeat condition, no significant effects were observed for run (*F*_2, 104_ = 0.471, *p* = .625, *η*^2^_p_ = .009), group (*F*_1, 52_ = 0.763, *p* = .386, *η*^2^_p_ = .014), or their interaction (*F*_2, 104_ = 0.572, *p* = .566, *η*^2^_p_ = .011). Similarly, in the visual attention condition, neither the effect of run (*F*_2, 104_ = 0.732, *p* = .483, *η*^2^_p_ = .014), group (*F*_1, 52_ = 0.057, *p* = .812, *η*^2^_p_ = .001), nor their interaction (*F*_2, 104_ = 0.226, *p* = .798, *η*^2^_p_ = .004) reached significance. These results suggest that NF training did not lead to systematic changes in averaged BOLD activation levels within the ROI, which differs from the result of Zhang et al. (2023).

Beyond univariate activation analysis, we performed an offline MVPA to examine whether NF training modulated the distinctiveness of neural representations for interoceptive and exteroceptive attention within the right insula. Offline classification accuracy between these states in the ROI was calculated for each run, including the attention decoder training task, NF regulation run 1, run 2, and run 3. We conducted a repeated-measures ANOVA on the classification accuracy, with run (Training, Regulation 1, 2, 3) as a within-subject factor and group (NF, Sham) as a between-subject factor (Fig. 4C). Results revealed a marginally significant run × group interaction (*F*_3, 156_ = 2.708, *p* = .047, *η*^2^_p_ = .050), while the main effects of run (*F*_3, 156_ = 1.119, *p* = .343, *η*^2^_p_ = .021) and group (*F*_1, 52_ = 1.774, *p* = .189, *η*^2^_p_ = .033) were not significant. To explore this interaction, we performed paired t-tests comparing classification accuracy between the decoder training task and each regulation run separately for each group. In the NF group, classification accuracy in Run 1 significantly outperformed that of the decoder training task (*t*_27_ = -3.034, *d* = -0.573 95% CI = [-0.969, -0.168], Bonferroni-corrected *p* = .015). However, comparisons between the decoder training task and Runs 2 and 3 did not reach significance with Bonferroni correction while showing similar trends (*t*_27_ = -2.024, *d* = -0.382, 95% CI = [-0.763, 0.005], corrected *p* = .159; *t*_27_ = -2.478, *d* = -0.468, 95% CI = [-0.855, -0.074], corrected *p* = .060). In the Sham group, no significant differences were observed across runs (*t*_25_s < |0.961|, *d*’s < 0.188, corrected-*p*’s = 1), suggesting that sham feedback did not lead to systematic pattern changes. These results suggest that while overall insula activation levels remained unchanged, NF training potentially modulated neural patterns associated with interoceptive and exteroceptive attention, particularly during early training. The absence of a similar effect in the Sham group implies that these changes were specific to contingent NF rather than general task repetition.

We next checked the real-time (online) decoder to test for a common spatial configuration across participants within the right-insula ROI. For each participant, we extracted the ROI weight vector from the linear SVM retrained at the end of regulation Run 2 (i.e., the decoder driving feedback in Run 3), back-projected it to native space to form a weight map (positive values favouring interoceptive attention; negative favouring exteroceptive), and entered these maps into group analyses. Within-group one-sample t-tests (uncorrected *p* < .005, k > 5) yielded no surviving clusters for either positive or negative direction in either the NF or Sham group; between-group two-sample tests were likewise non-significant. Thus, despite NF strengthening participants’ ability to instantiate the target state, informative voxels showed limited cross-participant overlap, so we refrain from further anatomical localisation and interpret the decoding gain as enhanced within-subject pattern discriminability rather than a shared group-level topography.

### 3.3 Changes in interoceptive accuracy between the groups

We next investigated whether training participants to discriminate insula activation patterns between heartbeat and visual attention would enhance interoceptive accuracy outside the MRI scanner. To this end, we compared interoceptive accuracy and confidence before (pre) and after (post) NF training in both groups. Repeated-measures ANOVAs were conducted with session (pre vs. post) as a within-subject factor and group (NF vs. Sham) as a between-subject factor.

**Fig. 5.**
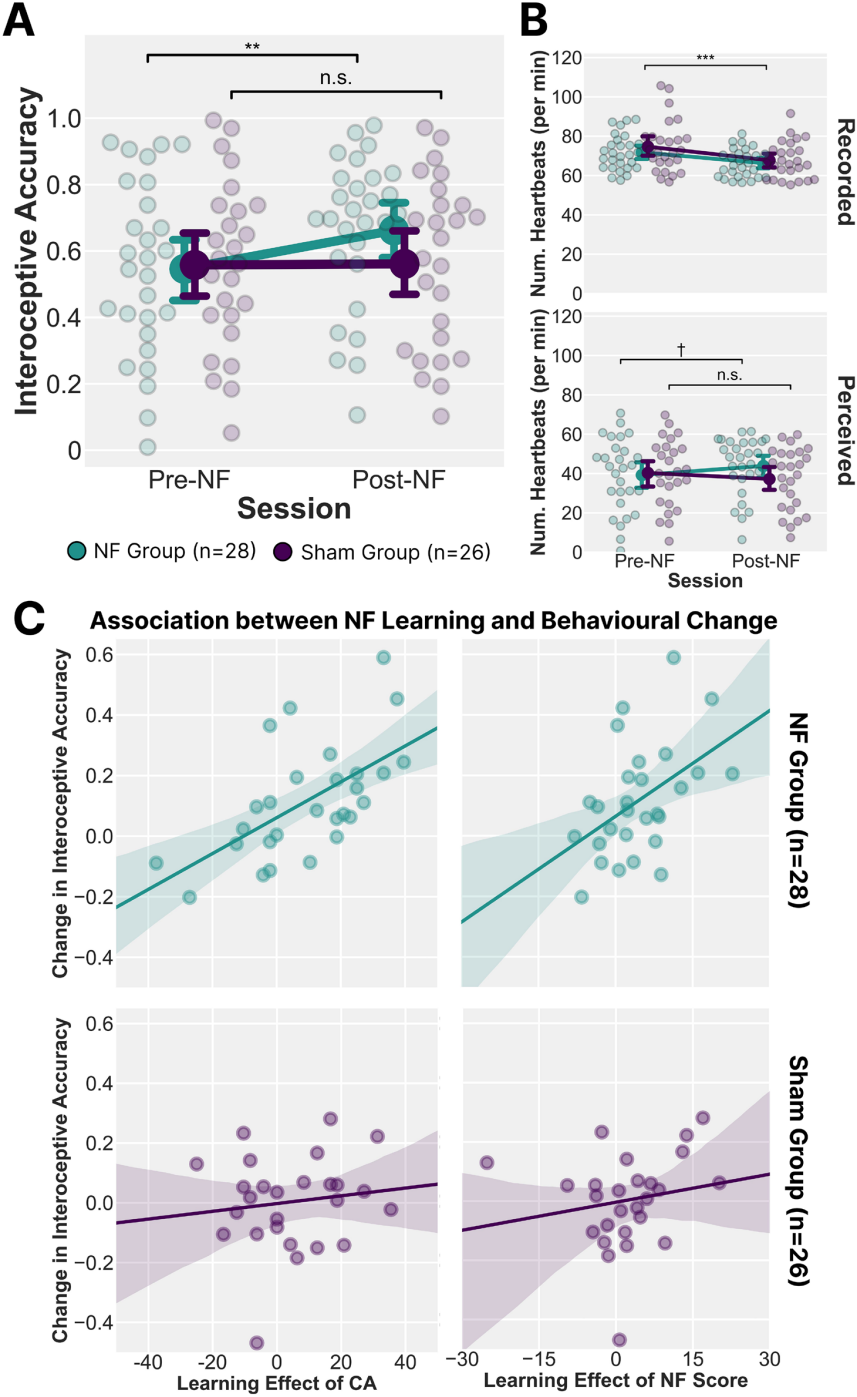
Changes in Interoceptive Accuracy and Relationship with Neurofeedback Learning. (A) Interoceptive accuracy before (Pre-NF) and after (Post-NF) neurofeedback (NF) training. A significant session × group interaction was observed, with the NF group (n = 28) showing improved accuracy post-training, while the Sham group (n = 26) showed no significant change. The main effect of the session was not significant. (B) Comparison of recorded and perceived heartbeats per minute across sessions. While actual heart rate decreased similarly in both groups, only the NF group exhibited an increase trend in perceived heartbeat count post-training, demonstrating improved heartbeat perception. (C) Correlation between NF learning effects and changes in interoceptive accuracy. In the NF group, greater improvements in interoceptive accuracy were significantly associated with higher NF scores and classification accuracy (CA), whereas no such relationship was observed in the Sham group. Error bars indicate mean ± SEM. ***: *p* < .001; **: *p* < .01; †: *p* < .10; n.s.: *p* > .10.

For interoceptive accuracy, there was a significant main effect of session (*F*_1, 52_ = 6.412, *p* = .014, *η*^2^_p_ = .110) and a session-by-group interaction (*F*_1, 52_ = 5.733, *p* = .020, *η*^2^_p_ = .099), though no significant main effect of group (*F*_1, 52_ = 0.491, *p* = .487, *η*^2^_p_ = .009) (Fig. 5A). Post hoc comparisons revealed that only the NF group exhibited a significant increase in accuracy from pre- to post-training (*t* = 3.550, *d* = 0.466, corrected-*p* = .005), while the Sham group showed no significant change (*t* = 0.096, *d* = 0.013 corrected-*p* = 1). A parallel session × group interaction was observed for interoceptive confidence (*F*_1, 52_ = 5.113, *p* = .028, *η*^2^_p_ = .090), though neither group (*F*_1, 52_ = 0.948, *p* = .350, *η*^2^_p_ = .006) nor session (*F*_1, 52_ = 0.425, *p* = .517, *η*^2^_p_ = .008) showed a significant main effect. Post hoc comparisons did not detect a significant session effect in either group after multiple-comparison corrections (NF: *t* = 2.331, *d* = 0.365, corrected-*p* = .142; Sham: *t* = 0.894, *d* = 0.145, corrected-*p* = 1). However, a simple main effect analysis indicated that only the NF group showed a significant increase in confidence (*F* = 5.681, *p* = .024).

Given that interoceptive accuracy is calculated based on both actual and perceived heartbeats, we examined whether changes in perceived heartbeat count contributed to the observed accuracy improvement (Fig. 5B). Heart rate (actual beats per minute) decreased from pre- to post-training (Pre: NF group = 71.766 ± 8.975, Sham group = 74.661 ± 13.240, Post: NF group = 66.243 ± 7.132, Sham group = 67.700 ± 9.347). A repeated-measures ANOVA indicated a significant session effect (*F*_1, 52_ = 80.562, *p* < .001, *η*^2^_p_ = .608), though neither the main effect of group (*F*_1, 52_ = 0.704, *p* = .405, *η*^2^_p_ = .013) nor the session × group interaction (*F*_1, 52_ = 1.069, *p* = .306, *η*^2^_p_ = .020, respectively) was significant. Conversely, perceived heartbeats per minute exhibited a significant session × group interaction (*F*_1, 52_ = 5.171, *p* = .027, *η*^2^_p_ = .090) (Pre: NF group = 39.228 ± 18.486, Sham group = 40.292 ± 16.988; Post: NF group = 43.855 ± 14.824, Sham group = 37.230 ± 15.657). The main effect of the session (*F*_1, 52_ = 0.214, *p* = .645, *η*^2^_p_ = .004) and group (*F*_1, 52_ = 0.443, *p* = .509, *η*^2^_p_ = .008) was not significant. While post hoc comparisons did not reveal significant session-dependent improvement in either the NF group (*t* = 1.972, *d* = 0.280, corrected-*p* = .323) or Sham group (*t* = -1.258, *d* = -0.185, corrected-*p* = 1), a simple main effect analysis suggested a trend toward significance for session in the NF group (*F* = 3.890, *p* = .054). Overall, these findings indicate that the NF-induced improvement in interoceptive accuracy in the NF group was primarily driven by an improvement in heartbeat detection, rather than changes in heart rate itself.

### 3.4 Greater insula modulation via neurofeedback is linked to greater improvement in heartbeat perception

Given that only the NF group exhibited a significant learning effect in insula regulation, along with a corresponding improvement in heartbeat perception, we next examined whether these two outcomes were associated at an individual level. Specifically, we calculated Δ interoceptive accuracy for each participant by subtracting their pre-training score from their post-training score. We then computed Pearson’s correlation coefficients between Δ interoceptive accuracy and each of the two learning effect indices—one based on the NF score and the other on CA—separately for the NF and Sham groups (Fig. 5C).

Among NF participants (n = 28), Δ interoceptive accuracy correlated significantly with both the learning effect of the NF score (*r* = .466, 95% CI = [.112, .715], *p* = .012) and the learning effect of CA (*r* = .602, 95% CI = [.296, .796], *p* < .001). These findings indicate that individuals who better modulated insula-based attentional states (as indexed by NF score and CA) also showed greater improvement in heartbeat perception. By contrast, no significant correlations were found in the Sham group (n = 26) for either the true (but unobservable to the participants) NF score (*r* = .180, 95% CI = [-.222, .531], *p* = .378) or CA (*r* = .128, 95% CI = [-.272, .491], *p* = .532). Importantly, a z-test revealed that the correlation between Δ interoceptive accuracy and the learning effect of CA was significantly stronger in the NF group than in the Sham group (*z* = 1.780, *p* = .038), with a similar significance trend for the NF score (*z* = 1.302, *p* = .096). Together, these results demonstrate a clear within-individual link between neural pattern learning and behavioural gains in interoceptive accuracy, specifically in the contingent NF group. This suggests that participants who successfully learned to modulate insula activation patterns were also those who showed the greatest improvement in heartbeat perception, supporting the idea that targeted NF training can facilitate functional gains in interoceptive processing.

### 3.5 Absence of NF training effect on emotional appraisal

Finally, we evaluated whether NF training altered participants’ behavioural and neural responses to negatively valenced images. On the behavioural side, a repeated-measures ANOVA on subjective valence ratings revealed a significant main effect of valence (*F*_1,51_ = 479.807, *p* < .001, *η*^2^_p_ = 0.904), indicating that negative images were consistently rated as less pleasant than neutral images. However, there were no significant main effects of session (*F*_1,51_ = 0.490, *p* = .487, *η*^2^_p_ = 0.010) or group (*F*_1,51_ = 1.613, *p* = .210, *η*^2^_p_ = 0.031). Furthermore, no significant interactions were observed, including the session × group interaction (*F*_1,51_ < 0.001, *p* = .982, *η*^2^_p_ < 0.001), the valence × group interaction (*F*_1,51_ = 0.486, *p* = .489, *η*^2^_p_ = 0.009), the session × valence interaction (*F*_1,51_ = 1.267, *p* = .266, *η*^2^_p_ = 0.024), or the three-way interaction involving valence (*F*_1,51_ = 2.196, *p* = .145, *η*^2^_p_ = 0.041) (see Supplementary Fig. S1A). These results suggest that NF training did not modulate subjective emotional appraisal across sessions. Thus, while NF training successfully enhanced heartbeat perception, it did not significantly influence the subjective evaluation of emotional stimuli under the present experimental conditions. Moreover, as detailed in the Supplementary Materials, we found no evidence that the training modulated activation across sessions in either the amygdala or insula.

## 4 Discussion

In this study, we used real-time NF based on right insula activity to train participants to discriminate and control patterns of interoceptive (heartbeat-focused) and exteroceptive (visual-focused) attention. We investigated the effects of this training by comparing an NF group that received contingent feedback with a Sham group that received non-contingent feedback. Our results showed that the NF group exhibited within-group improvements in the regulation performance from early to late runs, as well as a significant increase in heartbeat perception accuracy from pre- to post-training. These improvements were not observed in the Sham group, although a direct group comparison did not reach significance. Importantly, only in the NF group, the degree of successful regulation positively correlated with changes in interoceptive accuracy, suggesting that better insular modulation predicted greater gains in heartbeat detection. Meanwhile, neither group showed significant alterations in subjective emotional appraisal or amygdala activation in response to negative stimuli, indicating that a single short-term NF session may not suffice to induce noticeable changes in emotional reactivity.

A central finding of this study was that the NF group demonstrated a significant pre-to-post increase in heartbeat detection. Notably, this group-level improvement was driven by substantial individual-level effects: participants who successfully modulated their insular activation patterns achieved greater gains in interoceptive accuracy. Our NF paradigm targeted dissociating and reinforcing distinct activation patterns associated with interoceptive and exteroceptive attention within the right insula, a region central to both bodily awareness and salience detection (Haruki & Ogawa, 2021; Haufler et al., 2022; Kalhan et al., 2022; Simmons et al., 2013; Sridharan et al., 2008). From a neurocomputational standpoint, the insula has been proposed to implement a predictive model of the body’s internal state (Barrett & Simmons, 2015). Interoceptive accuracy, in this view, depends on the precision (gain) assigned to prediction errors within this model, which is enhanced by selectively attending to internal signals (Ainley et al., 2016; Haruki & Ogawa, 2024). Therefore, within this predictive-processing framework, our findings could be interpreted as an increase in precision arising from greater separation between interoceptive and exteroceptive attention states, thereby supporting improved heartbeat detection. Although visceral afferents are first relayed in brainstem nuclei (e.g., the nucleus tractus solitarius and parabrachial complex) and engage subcortical integrative hubs such as the periaqueductal grey (Saper, 2002), volitional modulation of precision may be implemented cortically—via attentional gain control—where ascending signals meet predictions (Barrett & Simmons, 2015; Seth et al., 2012); on this view, the insula is a plausible, albeit provisional, locus for such optimisation. Nonetheless, further studies are needed to validate this proposed mechanism of insula-mediated attentional modulation.

Notably, this improvement occurred despite the absence of explicit instructions to enhance heartbeat detection, suggesting that the ability to modulate insular activity in a task-relevant manner generalised to behavioural measures of heartbeat perception. This is particularly noteworthy given that other common interventions for enhancing bodily awareness, such as mindfulness or other forms of meditation, have demonstrated limited efficacy in improving heartbeat detection performance (Schwartz et al., 2025; Treves et al., 2019). Beyond contemplative practices, cardiac-feedback training programmes that directly target heartbeat perception have also been reported (Meyerholz et al., 2019). However, the apparent gains are difficult to interpret: manipulations using false cardiac feedback can similarly increase behavioural scores, and a follow-up study using a different behavioural task did not reproduce the effect (Rominger et al., 2021). These limitations highlight the potential of the pattern-based NF as a complementary, state-specific approach. Importantly, our finding contrasts with a previous study employing simpler averaged BOLD signal-based feedback, which, despite similar instructional frameworks, failed to elicit significant pre-to-post improvements in interoceptive accuracy (Zhang et al., 2023). The discrepancy between these outcomes underscores the methodological advantage of pattern-based classification approaches, which provide feedback based on state-specific neural signatures rather than broad amplitude-based changes that may lack functional specificity (deBettencourt et al., 2015; Watanabe et al., 2017). It should be noted that while our method employs a decoder, it is distinct from Decoded Neurofeedback (DecNeF), which typically relies on implicit learning (Amano et al., 2016; Koizumi et al., 2016; Shibata et al., 2019). Our choice of an explicit instructional framework was deliberate, as our primary goal was to test whether the volitional control over attentional shift between interoceptive and exteroceptive attention states could be trained to improve behaviour (i.e., heartbeat perception and emotional recognition).

Despite the observed individual-level association between NF regulation success and behavioural improvement, the absence of statistically robust group-level differences in the NF score limits the extent to which volitional control over right insula activation can be inferred. While only the NF group exhibited significant within-group improvements in regulation performance between earlier runs (runs 1 and 2) and the final run (run 3), a direct comparison with the Sham group—whose feedback was non-contingent—did not reveal statistically robust group-level differences. This lack of significance at the group level may stem from variability in individual learnability, as prior meta-analysis suggests that an average of about 38% participants exhibit NF inefficiency (Haugg et al., 2020), struggling to achieve meaningful regulation even under optimal conditions (Chiew et al., 2012; Ramot et al., 2016; Robineau et al., 2017; Scharnowski et al., 2012). Moreover, the artificially improving feedback provided to the Sham group may have influenced their performance, aligning with evidence that placebo effects can significantly impact NF outcomes (Thibault et al., 2018). These findings highlight the challenge of detecting group-level effects in complex fMRI NF tasks, underscoring the need to understand a better experimental design and individual variability in factors such as motivation and strategy use that may drive successful regulation (Haugg et al., 2021; Kadosh & Staunton, 2019). Promisingly, our offline MVPA suggests that the NF group exhibited a systematic modulation of insula activation patterns associated with interoceptive and exteroceptive attention, as reflected in an increase in decoding accuracy during training runs.

We also examined whether real-time NF training, which dissociated interoceptive and exteroceptive attention, influenced emotional responses to negative stimuli. However, neither subjective valence ratings nor bilateral amygdala activity showed significant changes, suggesting that a single session of NF targeting the insula alone was insufficient to alter emotional processing. This contrasts with mindfulness research, where prolonged training in bodily awareness and attentional control has been associated with reduced emotional reactivity and decreased amygdala activation (Desbordes et al., 2012; Kral et al., 2018). Given that our NF protocol involved only a single, 2-hour session without explicit emotion regulation instructions, it is likely that the intervention did not sufficiently engage the broader networks involved in affect regulation, such as prefrontal–amygdala interactions (Doll et al., 2016). While insula-based NF may enhance interoceptive accuracy, its effects on emotional processing may require additional cognitive components, such as explicit reappraisal strategies or prolonged training.

Nevertheless, the enhancement of interoceptive accuracy itself holds significant clinical potential, even in the absence of immediate emotional changes. While our study did not measure clinical or well-being outcomes, the observed enhancement of interoceptive accuracy has important implications for understanding psychiatric conditions characterised by dysfunction in interoceptive processing. For example, deficits in perceiving and interpreting bodily states are prominent features in affective disorders such as depression and alexithymia (Trevisan et al., 2019; Zhou et al., 2024). Our finding that targeted, pattern-based NF training can improve heartbeat perception points to a specific mechanism—the recalibration of interoceptive attention by separating its neural activation pattern from exteroceptive one—that could be a potential for future therapeutic interventions. Therefore, exploring this paradigm as a tool to directly probe and potentially improve the disturbances in interoceptive processing in these conditions presents a valuable direction for future clinical research.

Several limitations should be considered when interpreting the present findings. First, our recruitment procedure was sequential and not truly randomised, which gave rise to a small but statistically significant age difference between the NF and Sham groups. Although age was not significantly correlated with NF success, we cannot entirely rule out the possibility that this demographic difference influenced the results. Second, the relatively modest sample size and the single-session NF protocol limit the generalisability of our findings, particularly concerning long-term effects. Future studies should examine whether extended training enhances the robustness of NF-induced changes. Relatedly, because our NF training alternated interoceptive and exteroceptive trials for SVM calibration and drift control, attentional alternation itself cannot be ruled out entirely as a contributing factor; future work could test a unidirectional variant to isolate interoceptive-specific learning effects. Third, our protocol targeted the right insular cortex, yet interoceptive processes likely involve broader networks. A distributed, network-based NF approach could provide a more comprehensive understanding of how interoceptive processing and emotion regulation interact at the neural level. Fourth, this study did not include a self-report measure to assess broader changes in body awareness, which would be a valuable addition to future research to complement objective performance tasks. Fifth, due to a technical failure in data logging, the subjective intensity ratings from the NF training were irretrievably lost for a majority of participants (36 out of 54). This unfortunately precludes a more detailed exploration of the relationship between perceived intensity and regulation performance. Finally, the blinding procedure warrants consideration. While participants were unaware of the existence of a Sham group, which likely minimised their expectancy effects, the experimenters were not blind to group allocation, raising the possibility of subtle experimenter effects. Furthermore, we did not include a formal assessment, such as a post-experiment questionnaire, to confirm whether participants were successfully blinded.

## 5 Conclusion

This study demonstrates that real-time fMRI NF targeting the right insula can enhance heartbeat perception, with individual differences in regulation success predicting behavioural improvements. While group-level effects on NF scoring were not statistically robust, offline MVPA results suggest that only the NF group exhibited systematic modulation of the insula activation patterns during regulation runs, suggesting state-specific learning. The intervention did not significantly alter emotional responses, suggesting that short-term insula-based NF alone is insufficient to modulate affective processing. These findings highlight the potential of NF for enhancing interoceptive awareness but underscore the need for extended training or broader network-based approaches to maximise its effects.

## Supplementary Material

Supplementary Material

## Data Availability

Anonymised functional and anatomical data in BIDS format are publicly available at https://osf.io/58mgx/. Custom code used to run the main experiment will be made available, except for components subject to copyright restrictions.
