# Supplementary material for: Real-time fMRI neurofeedback boosts heartbeat perception by modulating insula activation pattern during interoceptive attention
Source: Imaging Neurosci (Camb). 2025 Sep 9;3:IMAG.a.142. doi: 10.1162/IMAG.a.142 (PMC12421695; doi:10.1162/IMAG.a.142)
Supplement: Supplementary Material [file IMAG.a.142_supp.pdf]

Supplementary materials

"Real-time fMRI neurofeedback boosts heartbeat perception by modulating insula activation pattern during interoceptive attention"

by Yusuke Haruki, Yuxiang Yang, Keisuke Suzuki, Hiroshi Imamizu, and Kenji Ogawa

Brain Activation during the Emotion Appraisal Task

In the main text, we found that the neurofeedback (NF) training did not modulate subjective emotional appraisal across sessions (Supplementary Figure S1A). To further investigate whether the training modulated neural responses to emotional stimuli, we analysed BOLD activity in the bilateral amygdala and insula during the pre- and post-training emotional appraisal task, given their central role in affective processing. For each region, a 2 (Valence: Negative, Neutral) × 2 (Session: Pre, Post) × 2 (Group: NF, Sham) mixed-design ANOVA was conducted. The results for each region are detailed below.

In the right amygdala, there was a significant main effect of valence ($F_{1, 51} = 9.489, p = .003, \eta^2_p = .157$), indicating stronger activation in response to negative images (Supplementary Figure S1B). However, no significant main effects of session ($F_{1, 51} = 1.408$, $p = .241$, $\eta^2_p = .027$) or group ($F_{1, 51} = 0.895$, $p = .349$, $\eta^2_p = .017$) were observed. Additionally, none of the interaction terms reached significance, including the session × group interaction ($F_{1, 51} = 0.047$, $p = .828$, $\eta^2_p < .001$), the valence × group interaction ($F_{1,51} = 0.095$, $p = .759$, $\eta^2_p = 0.002$), the session × valence interaction ($F_{1,51} < 0.001$, $p = .984$, $\eta^2_p < 0.001$), and the three-way interaction ($F_{1,51} = 0.468$, $p = .497$, $\eta^2_p = 0.009$). These results indicate that while the right amygdala showed greater activation to negative stimuli, no significant improvement induced by NF training was observed. A similar trend was observed in the left amygdala, where there was a trend-level main effect of valence ($F_{1, 51} = 3.627$, $p = .062$, $\eta^2_p = .066$), suggesting slightly greater activation in response to negative images. However, there were no significant main effects of session ($F_{1, 51} = 2.570$, $p = .115$, $\eta^2_p = .048$) or group ($F_{1, 51} = 1.566$, $p = .216$, $\eta^2_p = .030$), nor any significant interactions. These included session × group ($F_{1, 51} = 0.654$, $p = .423$, $\eta^2_p = .013$), valence × group ($F_{1, 51} = 0.009$, $p = .924$, $\eta^2_p < .001$), session ×

valence ($F_{1, 51}$ = 0.058, p = .810, η^2_p = .001), and the three-way interaction ($F_{1, 51}$ = 0.101, p = .751, η^2_p = .002).

Next, we examined activity in the insula. The ANOVA for the right insula revealed no significant main effect of valence ($F_{1, 51}$ = 2.235, p = .141, η^2_p = .042), session ($F_{1, 51}$ = 0.390, p = .535, η^2_p = .008) or group ($F_{1, 51}$ = 0.206, p = .652, η^2_p = .004) (Supplementary Figure S1C). Additionally, none of the interaction terms reached significance: session × group ($F_{1, 51}$ = 0.894, p = .349, η^2_p = .017), valence × group ($F_{1, 51}$ = 0.265, p = .609, η^2_p = .005), session × valence ($F_{1, 51}$ = 0.463, p = .500, η^2_p = .009), and the three-way session × valence × group interaction ($F_{1, 51}$ = 0.098, p = .756, η^2_p = .002). A similar analysis of the left insula showed a significant main effect of valence ($F_{1, 51}$ = 6.188, p = .016, η^2_p = .108). There was a slight trend towards significance in the main effect of the session ($F_{1, 51}$ = 3.320, p = .074, η^2_p = .061). However, we found no significant main effect of the group ($F_{1, 51}$ = 0.214, p = .646, η^2_p = .004), nor any significant interactions. These included session × group ($F_{1, 51}$ = 0.310, p = .580, η^2_p = .006), valence × group ($F_{1, 51}$ = 0.132, p = .718, η^2_p = .003), session × valence ($F_{1, 51}$ = 0.350, p = .556, η^2_p = .007), and the three-way interaction ($F_{1, 51}$ = 0.155, p = .695, η^2_p = .003).

Taken together, these findings indicate that while NF training successfully enhanced heartbeat perception, it did not significantly modulate subjective emotional appraisal or amygdala reactivity to negative stimuli.

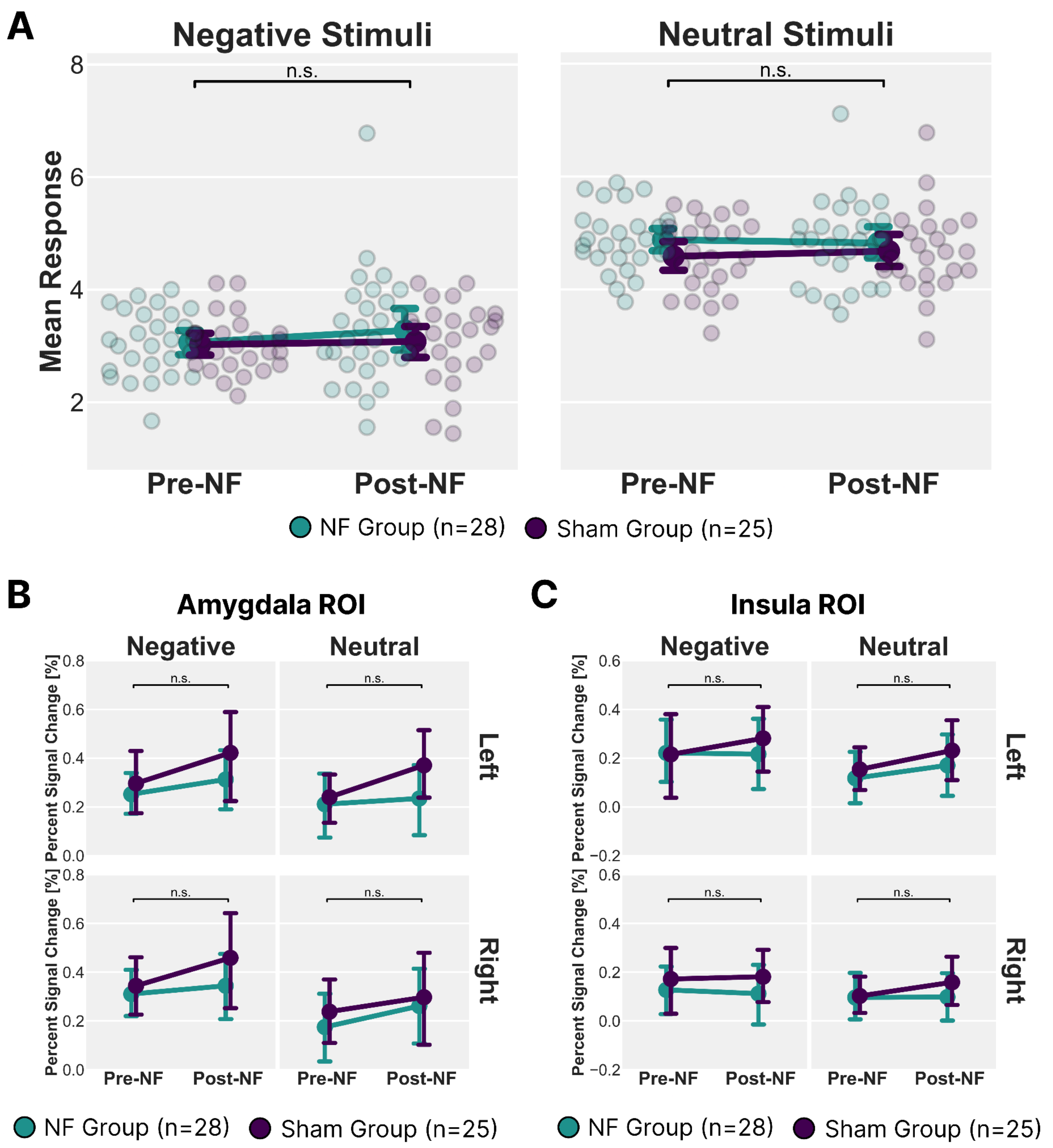

Supplementary Figure S1. Limited Effects of Neurofeedback Training on Emotional Processing. (A) Mean subjective valence ratings for negative and neutral stimuli before (Pre-NF) and after (Post-NF) neurofeedback (NF) training. No significant differences were observed across sessions or between the NF ($n = 28$) and Sham ($n = 25$) groups, indicating that NF training did not modulate subjective emotional appraisal. (B) Percent signal change in the left and right amygdala, the region of interest (ROI) for affective processing, during the presentation of negative and neutral stimuli. While the right amygdala showed a main effect of valence, with stronger activation for negative stimuli, no significant session or group effects were found in either hemisphere. (C) Percent signal change in the left and right insula

during the stimulus presentation. Though the left insula showed a main effect of valence, with stronger activation for negative stimuli, no significant session or group effects or their interactions were found in either hemisphere. One participant in the sham group was excluded from these analyses due to missing responses in Post-NF session. Error bars indicate mean ± SEM. n.s.: $p > .05$
